# On the Diversification of the Translation Apparatus across Eukaryotes

**DOI:** 10.1155/2012/256848

**Published:** 2012-05-14

**Authors:** Greco Hernández, Christopher G. Proud, Thomas Preiss, Armen Parsyan

**Affiliations:** ^1^Division of Basic Research, National Institute for Cancer (INCan), Avenida San Fernando No. 22, Col. Sección XVI, Tlalpan, 14080 Mexico City, Mexico; ^2^Centre for Biological Sciences, University of Southampton, Life Sciences Building (B85), Southampton SO17 1BJ, UK; ^3^Genome Biology Department, The John Curtin School of Medical Research, The Australian National University, Building 131, Garran Road, Acton, Canberra, ACT 0200, Australia; ^4^Goodman Cancer Centre and Department of Biochemistry, Faculty of Medicine, McGill University, 1160 Pine Avenue West, Montreal, QC, Canada H3A 1A3; ^5^Division of General Surgery, Department of Surgery, Faculty of Medicine, McGill University Health Centre, Royal Victoria Hospital, 687 Pine Avenue West, Montreal, QC, Canada H3A 1A1

## Abstract

Diversity is one of the most remarkable features of living organisms. Current assessments of eukaryote biodiversity reaches 1.5 million species, but the true figure could be several times that number. Diversity is ingrained in all stages and echelons of life, namely, the occupancy of ecological niches, behavioral patterns, body plans and organismal complexity, as well as metabolic needs and genetics. In this review, we will discuss that diversity also exists in a key biochemical process, translation, across eukaryotes. Translation is a fundamental process for all forms of life, and the basic components and mechanisms of translation in eukaryotes have been largely established upon the study of traditional, so-called model organisms. By using modern genome-wide, high-throughput technologies, recent studies of many nonmodel eukaryotes have unveiled a surprising diversity in the configuration of the translation apparatus across eukaryotes, showing that this apparatus is far from being evolutionarily static. For some of the components of this machinery, functional differences between different species have also been found. The recent research reviewed in this article highlights the molecular and functional diversification the translational machinery has undergone during eukaryotic evolution. A better understanding of all aspects of organismal diversity is key to a more profound knowledge of life.

## 1. Protein Synthesis Is a Fundamental Process of Life

Proteins are one of the elementary components of life and account for a large fraction of mass in the biosphere. They catalyze most reactions that sustain life and play structural, transport, and regulatory roles in all living organisms. Hence, “translation,” that is, the synthesis of proteins by the ribosome using messenger (m)RNA as the template, is a fundamental process for all forms of life, and a large proportion of an organism's energy is committed to translation [1, 2]. Accordingly, regulating protein synthesis is crucial for all organisms. Indeed, many mechanisms to control gene expression at the translational level have evolved in eukaryotes [3]. These mechanisms have endowed eukaryotes with the potential to rapidly and reversibly respond to stress or sudden environmental changes [1, 2, 4]. Translational control also plays a crucial role in tissues and developmental processes where transcription is quiescent, or where asymmetric spatial localization of proteins is required, such as early embryogenesis, learning and memory, neurogenesis, and gametogenesis [5–10]. Moreover, recent global gene expression measurements have shown that the cellular abundance of proteins in mammalian cells is predominantly controlled at the level of translation [11, 12].

Eukaryotic translation is a sophisticated, tightly regulated, multistep process, the basic steps of which are conserved in all eukaryotes. It is performed by the ribosome together with multiple auxiliary “translation” factors (proteins) and is divided into four steps: initiation, elongation, termination, and recycling. These basic processes of translation were established experimentally in eukaryotes some decades ago, and many regulatory mechanisms have been subsequently elucidated [13, 14]. However, it was only recently that, with the use of powerful genome-wide sequencing, proteomics and bioinformatics-based technologies, a surprising diversity in components of the translation apparatus across eukaryotes was unveiled. In some cases, even functional differences between same molecules from different species have also been identified. Additionally, there is evidence that even the genetic code itself has continued to evolve in some phyla. These findings indicate that after eukaryotes emerged, the translational apparatus further evolved during eukaryotic diversification. In this article, we will review recent research revealing the diversification that the genetic code and many components of the translational machinery have undergone across eukaryotes.

## 2. Overview of the Translation Process in Eukaryotes

### 2.1. Initiation

**Figure 1:**
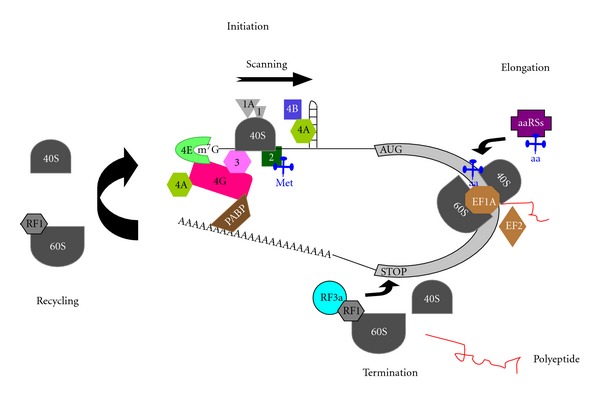
The general process of translation in eukaryotes. A typical eukaryotic mRNA is represented. The cap structure (m^7^G), the open reading frame (light gray box) and the poly(A) tail are depicted. During *Initiation*, most eukaryotic mRNAs are translated by the cap-dependent mechanism, which requires recognition by eIF4E (green crescent) complexed with eIF4G (red) and eIF4A (light green)—the so-called eIF4F complex—of the cap structure at the 5′ end. A 43S preinitiation complex (consisting in a 40S ribosomal subunit (dark gray) loaded with eIF3 (pink), eIF1 and eIF1A (light grey), initiator Met-tRNA_i_^Met^ (blue clover), eIF2 (dark green), and GTP binds the eIF4F-mRNA complex and scans along the 5′-UTR of the mRNA to reach the start codon (usually an *AUG* triplet). During the scanning eIF4A, stimulated by eIF4B (dark blue), unwinds secondary RNA structure in an ATP-dependent manner. The poly A-binding protein (PABP, dark brown) binds both the poly(A) tail and eIF4G promoting mRNA circularization. *Elongation* is assisted by elongation factors eEF1A and eEF2 (light brown). During this step, aminoacyl-tRNA synthetases (aaRSs, purple) catalyze the binding of amino acids (aa) to cognate tRNAs. *Termination* is mediated by the release factors eRF1 (gray) and eRF3 (light blue) and happens when a termination codon (*STOP*) of the mRNA is exposed in the A-site of the ribosome. In this step, the completed polypeptide (red) is released. During *Recycling*, which is required to allow further rounds of translation, both ribosomal subunits dissociate from the mRNA. eRF1 remains associated with the posttermination complexes after polypeptide release.

The aim of the initiation step is both to ensure the recruitment of the mRNA to the ribosome and the positioning the ribosome in the proper frame at the start codon, which is achieved in a set of steps mediated by eukaryotic initiation factors (eIF). For most eukaryotic mRNAs, this happens by the so-called cap-dependent mechanism (Figure 1) [15–18]. It begins with the dissociation of the ribosome into its 60S and 40S subunits by eIF6. Free 40S subunit, which is stabilized by eIF3, eIF1, and eIF1A, binds to a ternary complex (consisting of eIF2 bound to an initiator Met-tRNA_i_^Met^ and GTP) to form a 43S preinitiation complex. On the other hand, the cap structure (m^7^GpppN, where N is any nucleotide) of the mRNA is recognized by eIF4E in complex with the scaffold protein eIF4G. Then, the 43S preinitiation complex is recruited to the 5′ end of the mRNA, a process that is coordinated by eIF4E through its interactions with eIF4G and the 40S ribosomal subunit-associated eIF3. The ribosomal complex then scans in a 5′*→* 3′ direction along the 5′-untranslated region (UTR) through interactions with the eIF4G-bound RNA helicase eIF4A and eIF4B to reach the start codon, usually an AUG. During scanning, eIF4B stimulates the activity of eIF4A which unwinds secondary RNA structures in the mRNA. eIF1, eIF1A, and eIF5 assist in the positioning of the 40S ribosomal subunit at the correct start codon so that eIF2 can deliver the anti-codon of the initiator Met-tRNA_i_^Met^ as the cognate partner for the start codon, directly to the peptidyl (P)-site of the 40S ribosomal subunit. Once the ribosomal subunit is placed on the start codon, a 48S pre-initiation complex is formed. Then, eIF5 promotes GTP hydrolysis by eIF2 to release the eIF proteins. Finally, the GTPase eIF5B is required for the joining of the 60S ribosomal subunit to the 40S subunit to form an 80S initiation complex. The poly A-binding protein (PABP) is able to interact with the 3′-poly(A) tail and eIF4G promoting circularization of the mRNA and increasing the efficiency of subsequent rounds of initiation (Figure 1) [15–20].

In the case of some viral and cellular mRNAs, 5′-UTR recognition by the 40S ribosomal subunit happens without involvement of eIF4E and is, instead, driven by RNA structures located in *cis* within the mRNA itself. Such structures are operationally defined as internal ribosome entry site (IRES) and are located in the proximity of the start codon ([21–23]; Martinez-Salas et al. this issue).

### 2.2. Elongation

After initiation, the 80S ribosome is assembled at the start codon of the mRNA containing a Met-tRNA_i_^Met^ in the P-site. Then, elongation takes place (Figure 1); this is the process of decoding codons and formation of peptide bonds sequentially to add amino acid residues to the carboxy-terminal end of the nascent peptide [16, 24–26]. This process is assisted by elongation factors (eEF) and involves four major steps. (1) Formation of the ternary complex eEF1A·GTP·aminoacyl-tRNA and delivery of the first elongator aminoacyl-tRNAs to an empty ribosomal tRNA-binding site called the A-(acceptor) site. It is in the A-site where codon/anticodon decoding takes place. (2) Interaction of the ribosome with the mRNA-tRNA. This duplex activates eEF1A·GTP hydrolysis and guanine nucleotide exchange on eEF1A. (3) Peptide bond formation then occurs between the P-site peptidyl-tRNA and the incoming aminoacyl moiety of an A-site aminoacyl-tRNA. This reaction is catalyzed by the peptidyl transferase center of the 60S ribosomal subunit, and the products comprise of a new peptidyl-tRNA that is one amino acid residue longer and a deacylated (discharged) tRNA. (4) Binding of eEF2·GTP and GTP hydrolysis promotes the translocation of the mRNA such that the deacylated tRNA moves to the E-(exit) site, the peptidyl-tRNA is in the P-site, and the mRNA moves by three nucleotides to place the next mRNA codon into the A-site. The deacylated tRNA in E-site is then ejected from the ribosome. The whole process is repeated along the mRNA sequence until a stop codon is reached and the process of termination is initiated [16, 24–26].

### 2.3. Termination

Translation termination is mediated by two polypeptide chain-release factors, eRF1 and eRF3 (Figure 1). When any of the termination codons (UAA, UAG, and UGA) is exposed in the A-site, eRF1 recognizes the codon, binds the A-site, and triggers the release of the nascent polypeptide from the ribosome by hydrolysing the ester bond linking the polypeptide chain to the P-site tRNA. This reaction leaves the P-site tRNA in a deacylated state, leaving it to be catalyzed by the peptidyl transferase center of the ribosome. eRF1 recognizes stop signals and functionally acts as a tRNA-mimic, whereas eRF3 is a ribosome- and eRF1-dependent GTPase that, by forming a stable complex with eRF1, stimulates the termination process [16, 27, 28].

### 2.4. Recycling

In the recycling step, both ribosomal subunits are dissociated, releasing the mRNA and deacetylated tRNA, so that both ribosomal subunits can be used for another round of initiation [16, 27, 28] (Figure 1). The closed-loop model proposes that, during translation, cross-talk occurs between both ends due to the circular conformation of the mRNA. According to this model, termination and recycling may not release the 40S ribosomal subunit back into the cytoplasm. Instead, this subunit may be passed from the poly(A) tail back to the 5′-end of the mRNA, so that a new round of initiation can be started [16, 27].

## 3. Divergence in the Genetic Code

**Figure 2:**
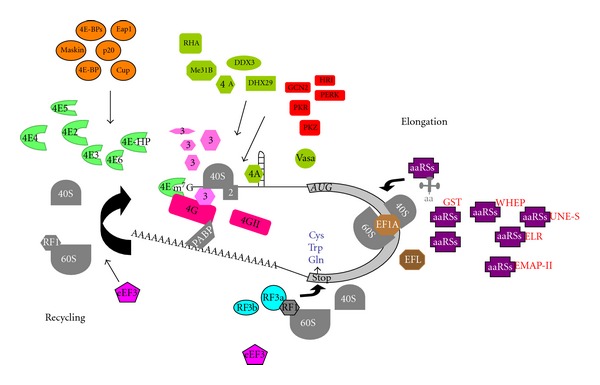
Diversity in the configuration of the translation apparatus across eukaryotes. The different components of the translation machinery that show diversity in different phyla are shown in colors. Components with some diversity that is not discussed here are depicted in gray. Several copies of eIF4E (green crescent) and eIF4G (red) have been found in plants, metazoan, and protists. In some cases, eIF4E cognates have evolved towards translational repressors (4E-HP is an example). Many 4E-binding proteins (orange) have been discovered in species from metazoan, fungi and protists. The subunit composition of eIF3 (pink) ranges from 5 to 13 nonidentical polypeptides in different phyla. There is, however, a core of five homolog subunits shared by most eukaryotes. Several RNA helicases (light green) from diverse organisms have been found to be involved in *Initiation*. A family of five kinases (*HRI*, *PERK*, *GCN2*, *PKR,* and *PKZ*, red) phosphorylate the alpha subunit of eIF2 to inhibit global translation under stress conditions. The presence of eIF2alpha kinases varies in different lineages. Different domains (red), such as *WHEP*, *EMAPII*, *ELR*, *GST,* and *UNE-S*, have been added to different aminoacyl-tRNA synthetases (*aaRSs*, purple) in distinct phyla of multicellular species. For *Elongation* to happen, a number of protist, algae and fungi (most of them unicellular organisms) lack eEF1A (light brown) and instead possess the related factor elongation factor-like (EFL, dark brown). For *Termination*, most organisms only contain a single eRF3 (light blue). In contrast, mammalian species express two eRF3s (viz. eRF3a and eRF3b). Ribosomes from all eukaryotes perform *Elongation* with eEF1A and eEF2. However, the yeast *S. cerevisiae* requires an additional essential factor, eEF3 (light purple), for *Elongation* to proceed. Genes encoding eEF3 have been found exclusively in many species of fungi. Evidence supports the notion that eEF3 activity promotes ribosome recycling. Variations to the “universal” genetic code, wherein the meaning of a “universal” codon is changed to a different one, exist in several species of in unicellular eukaryotes. Most codon variations are the reassignment of the stop codons UAG and UAA to glutamine, and the stop codon UGA to tryptophan or cysteine.

The deciphering of the genetic code in the early 1960's established one of the basic foundations of modern biology. Soon after, the essential universality of the genetic code was recognized, that is, the assignment of 20 amino acids to 64 codons and two punctuation marks (start and stop signals) is substantially the same for all extant forms of life on earth [29]. Nevertheless, variations to the “universal” genetic code, wherein the meaning of a “universal” codon is changed to a different one, have recently been uncovered in a wide range of bacteria, organelles, and the nuclear genome of eukaryotes, revealing that the genetic code is still evolving in some lineages [30–33]. In eukaryotes, deviations from the standard nuclear genetic code have arisen independently multiple times in unicellular organisms of five lineages, namely, ciliates, Diplomonads, fungi (in the genus *Candida* and some ascomycetes), polymastigid oxymonads, and green algae (in Dasycladales and Cladophorales) [30, 31, 33–39]. Most codon variations in eukaryotes are found to be the reassignment of the stop codons UAG and UAA to glutamine, and the stop codon UGA to tryptophan or cysteine (Figure 2). All reported code variations in ciliates, Diplomonads, and green algae belong to this kind. In contrast, *Candida *ambiguously utilizes the codon CUG (universally used for leucine) for both serine and leucine. The underlying mechanisms of codon reassignment are mutations in tRNA genes that affect decoding, RNA editing, or mutations in eRF1 [30, 31, 34–39].

The observation that the same codon reassignments have occurred independently in closely related species (within the yeasts, green algae, and ciliate taxa) supports the notion that these changes provide a selective advantage in similar ecological niches [30]. Whether there is a restriction for the genetic code to change in multicellular organisms is not known.

## 4. Diversity in the Initiation Step

### 4.1. Functional Divergence of eIF Proteins

While the fundamental principles of translation are well conserved across all forms of life, in eukaryotes the initiation step has undergone substantial increase in complexity as compared to prokaryotes [3, 22, 40–44]. Most evidence for molecular and functional diversification among the translation components has been found in the eIF4 proteins (Figure 2). Most eukaryotic phyla possess several paralog genes for members of the eIF4 families, with well-documented differential expression patterns and variable biochemical properties among paralogs of the same organism [45–72]. For eIF4E and eIF4G cognates, even evidence of physiological specialization has been found among both unicellular and multicellular organisms (Table 1). These findings support the hypothesis that in organisms with several paralogs, an ubiquitous set of eIF4 factors supports global translation initiation whereas other paralogs perform their activity in specific cellular processes [45]. In some cases, eIF4E cognates have evolved towards translational repressors. Class 2 eIF4Es are exemplified by eIF4E-homolog protein (4E-HP) in human, eIF4E-2 in mouse [63], eIF4E-8 in *Drosophila* [52, 58, 73], IF4 in *C. elegans *[74, 75], and nCBP in *A. thaliana *[76], and they can bind the 5′ cap structure of mRNA but do not bind eIF4G [58, 77], thereby acting as a translational repressors of mRNAs associated with it [73, 78]. Class 2 eIF4Es are widespread across metazoa, plants, and some fungi although absent in the model ascomycetes *S. cerevisiae *and *S. pombe* [46]. Since the *Arabidopsis* [76] and *Caenorhabditis *[74] orthologs promote translation of some mRNAs, it seems most likely 4E-HP diverged from a widespread ancestral eIF4E to form a translational repressor in metazoa [3]. A similar example is eIF4E-1B, which emerged only in vertebrates as a translational repressor of a subset of oocyte mRNAs [57, 59, 79], and *Leishmania* eIF4E-1, which under heat shock conditions binds to a *Leishmania*-specific 4E-BP and becomes translationally inactive [71]. In other cases, eIF4E cognates have evolved towards a new molecular function not related to translation. This is the case with *Trypanosoma *eIF4E-1 and eIF4E-2, which are essential nuclear and cytoplasmic proteins, respectively [49], and *Giardia* (eIF4E-2), which binds only to nuclear noncoding small RNAs [64]. However, it is also possible that this was an ancestral function of eIF4E [22, 40].

Whereas the need for distinct eIF4 proteins in different tissues may have been the driving force behind the evolution of various paralogs in multicellular organisms, in unicellular eukaryotes different paralogs may be differentially needed during distinct life stages [49]. Specific features of mRNA metabolism in some phyla also might have driven the evolution of eIF4Es in specific organisms, such as the use of different cap structures (usually mono- and trimethylated) in mRNAs from worms of the phylum Nematoda [50, 51, 54, 80], and flagellate protists of the order Kinetoplastida [49, 65, 66]. These mRNAs result from the *trans*-splicing process to produce mature mRNAs. 

Other eIFs have also undergone molecular diversification across eukaryotes, including the multisubunit eIF3 whose subunit composition ranges from 5 to 13 nonidentical polypeptides in different phyla [99], and eIF6 that is duplicated into two or three paralogs in plants [100]. However, the functional relevance of these phenomena (if any) is not known. 

### 4.2. Multiple RNA Helicases for Translation Initiation

The evolution of cap-dependent translation has led to a dependency on RNA helicase activity to unwind the 5′-UTR secondary structure during the scanning [22, 40]. The DEAD-box RNA helicase/ATPase eIF4A is the main helicase thought to perform this activity. Recently, other RNA helicases from diverse organisms have also been found to facilitate translation of specific mRNAs with structured 5′-UTRs (Figure 2). Such is the case of the mammalian, *Drosophila* and yeast DEAD-box helicases DDX3 and Ded1, as well as the human DExH-box helicases RHA and DHX29 [101–103]. In *Drosophila*, the DEAD-box helicase Vasa interacts with eIF5B and regulates the translation of *gurken* and *mei-P26* mRNAs. Evidence supports the idea that Vasa is a translational activator of specific mRNAs involved in germline development [6, 7]. In contrast, orthologs of the *Xenopus* helicase Xp54 (DEAD-box, DDX6-like helicases) in a spectrum of organisms, including *Drosophila* Me31B, *Saccharomyces *Dhh1, human rck/p54, and *Caenorhabditis* CGH-1 have been found to repress translation of stored mRNAs and promote aggregation into germplasm-containing structures [104].

Most RNA helicases involved in translation also play a variety of roles in other processes of RNA metabolism, including mRNA RNP assembly, RNA degradation, RNA export, and splicing [103]. This functional versatility of RNA helicases leads us to speculate that a wider diversity of other, yet unidentified, helicases might be involved in translation in all eukaryotes. This could be the case of the *Arabidopsis* eIF4F complex, which contains eIF4A in proliferating cells but different RNA helicases in quiescent cells [105]. Whether these helicases play a role in translation is not known.

### 4.3. Divergence in the Regulation of Initiation: Diversity of eIF4E-Binding Proteins

Almost twenty years ago, it was discovered that eIF4E is negatively regulated in mammalian cells by three related proteins, the eIF4E-binding proteins (4E-BPs) 1, 2, and 3. These proteins share with eIF4G the motif YXXXXL*ϕ* (where X is any amino acid and *ϕ* is a hydrophobic residue) that interacts with the convex dorsal surface of eIF4E, so binding of 4E-BPs to eIF4E precludes its association with eIF4G and represses cap-dependent translation [8, 106]. In the last years, a myriad of 4E-binding proteins has been discovered in species from distantly related taxa, including mammals, plants, *Drosophila*, *Caenorhabditis*, yeast [3, 8, 106], and *Leishmania* [71] (Figure 2). Interestingly, most 4E-BPs are phylogenetically unrelated to each other and control translation in disparate, species-specific processes, such as embryogenesis in *Drosophila*, neurogenesis in mammals, or pseudohyphal growth in yeast. Moreover, some 4E-BPs utilize non-canonical motifs to bind eIF4E. These observations support the idea that binding to eIF4E evolved independently in multiple taxonomic groups [3].

### 4.4. Divergence in the Regulation of Initiation: The Case of eIF4E Phosphorylation

In mammalian cells, the kinases ERK or p38MAPK phosphorylate and activate the MAPK-interacting kinases (Mnk1/2). Mnk interacts with the carboxy-terminal part of eIF4G to directly phosphorylate eIF4E on Ser-209. This phosphorylation appears to regulate the function of eIF4E although the precise consequences are unclear [107–110]. Mammals possess two Mnk genes (*MKNK1/2*) which in humans, but not mice, give rise to four Mnk isoforms by alternative splicing; these isoforms have distinct properties in terms of activity, regulation, and subcellular localization [111]. In *Drosophila*, the single Mnk orthologue, LK6, also phosphorylates eIF4E-1 at a serine residue corresponding to mammalian Ser-209, a phosphorylation that is critical for development and cell growth [112–115]. However, the effects of phosphorylation on eIF4E activity and its physiological relevance are different across eukaryotes. Indeed, a residue equivalent to Ser-209 is present in metazoan eIF4Es but is absent in different fungi, protists and plants ([67]; R. Jagus et al., this issue). Accordingly, Mnk is conserved among metazoans, but no Mnk ortholog exists in *S. cerevisiae *or plants, whose eIF4Gs lack a Mnk-binding domain ([67]; R. M. Patrick and K. S. Browning, this issue). Moreover, *Trypanosoma* eIF4E-3 [116] and *S. cerevisiae* eIF4E [117] are phosphorylated on residues which are not equivalent to mammalian Ser-209, and *S. cerevisiae* cells expressing a nonphosphorylatable version as sole source of eIF4E do not display any evident defect on global protein synthesis or cell growth [117]. These observations support the idea that eIF4E phosphorylation at Ser-209 by the MAPK-Mnk signaling pathway evolved only in metazoans and that, perhaps, alternative mechanisms regulate eIF4E in nonmetazoan eukaryotes [3].

### 4.5. Diversity in the Regulation of Initiation: The Case of eIF2alpha Phosphorylation

Under different stress conditions, general protein synthesis is inhibited through phosphorylation of the alpha subunit of eIF2 at Ser-51 by a family of kinases that are present in widely scattered lineages. They include the double-stranded RNA protein kinase (PKR) that is activated during viral infection, the heme-regulated inhibitor kinase (HRI) that is activated under heme deprivation or arsenite exposure, the PKR-like endoplasmatic reticulum kinase (PERK) that is activated by unfolded proteins in the lumen of the endoplasmic reticulum, and the general control nonderepressible 2 (GCN2) that is activated by uncharged tRNA and thus senses amino acid starvation [107, 118] (Figure 2). The presence of eIF2alpha kinases varies in different lineages; while GCN2 is present in all eukaryotes; PERK is found in only metazoans; HRI is found in vertebrates, the dipteran *Anopheles*, the fungi* Schizosaccharomyces,* and the echinoderm *Strongylocentrotus*; PKR is only found in vertebrates [53, 118]. Interestingly, in some teleost fishes, PKR has undergone further duplication into PKR and PKZ, which perhaps led teleost fishes to respond to an extended range of viral infections [119].

## 5. Diversity in the Elongation Step

### 5.1. Divergence in the Aminoacyl-tRNA Synthetases

The process of elongation is highly conserved among all forms of life [16, 24, 25]. Key molecules for elongation are aminoacyl-tRNA synthetases (aaRSs), which catalyze the aminoacylation reaction whereby an amino acid is attached to the cognate tRNA. aaRSs are the only components of the gene expression machinery that function at the interface between nucleic acids and proteins. Thus, by performing their activity, aaRSs establishes the fundamental rules of the universal genetic code and, thus, of translation. aaRSs constitute a family of 20 essential cellular enzymes that are grouped into two classes: class I, in which the aminoacylation domain has a Rossmann nucleotide-binding fold, and class II, in which this domain is a seven-stranded beta-sheet with flanking alpha-helices. The conservation of the genetic code suggests that aaRSs evolved very early before the emergence of the last universal common ancestor [120, 121].

Throughout evolution of multicellularity, different domains, such as the WHEP domain, the oligonucleotide binding fold-containing EMAPII domain, the tripeptide ELR (Glu-Leu-Arg), the glutathione S-transferase (GST) domain and a specialized amino-terminal helix (N-helix), have been progressively added to different aaRSs in distinct phyla (Figure 2). The tripeptide ELR and the EMAPII domain were incorporated simultaneously to TyrRSs in metazoans starting from insects; the WHEP domain is present in TrpRS only in chordates; a unique sequence motif, UNE-S, became fused to the C-terminal of SerRS of all vertebrates [120, 121]. In bilaterian animals, the glutamylRS and prolylRS were linked via WHEP domains giving rise to a bifunctional glutamyl-prolylRS [120, 121]. It was recently found that this fused enzyme is also present in the cnidarian *Nematostella*, which pushes the origin of glutamyl-prolylRS back to the cnidaria-bilaterian ancestor [122], and suggests that this enzyme further underwent fission in the nematode *C. elegans* where glutamylRS and prolylRS enzymes are separated. GlutamylRS and prolylRS are also separate in plants and fungi [120–122].

It has been found that the function of the aaRSs was either increased or impaired by the addition of the new domains. Whereas the WHEP domain regulates interaction of TrpRS with its cognate receptor, with MetRS this domain plays a tRNA-sequestering function. The Leu-zipper motif in ArgRS is important for the formation of multi aaRSs complex (MSC), which enhances channeling of tRNA to the ribosome. Moreover, different aaRSs play diverse roles in cellular activities beyond translation, such as stress response, plant and animal embryogenesis, cell death, immune responses, transcriptional regulation, and RNA splicing [120, 121, 123]. It was found that the incorporation of domains to aaRSs correlates positively with the increase in organism's complexity. For example, the number of aaRSs carrying the GST domain increases from two in fungi to four in insects, to five in fish, and six in humans [121]. Thus, it has been proposed that the newly fused aaRSs domains triggered the appearance of new biological functions for these proteins in different lineages and that the fusion of domains to aaRSs could have played an important part in expanding the complexity of newly emerging metazoan phyla [121].

### 5.2. Divergence in Elongation Factors

eEF1A plays a critical role in translation. It binds and delivers aa-tRNAs to the A-site of ribosomes during the elongation step. Because homologs of this essential protein occur in all domains of life, it was thought to exist in all eukaryotes. Strikingly, a recent genome-wide survey revealed that a number of lineages lack eEF1A and instead possess a related factor called elongation factor-like (EFL) protein that retains the residues critical for eEF1A function [124] (Figure 2). It was later found that EFL-encoding species are scattered widely across eukaryotes and that *eEF1A* and *EFL* genes display mutually exclusive phylogenetic distributions. Thus, it is assumed that eEF1A and EFL are functionally equivalent [124–132]. Since EFL is present only in eukaryotes, it is thought that eEF1A is ancestral to all extant eukaryotes and that a single duplication event in a specific lineage gave rise to EFL. EFL genes were then spread to other lineages via multiple independent lateral gene transfer events, where EFL took over the original eEF1A function resulting in secondary loss of the endogenous eEF1A. It is thought that both genes coexisted for some time before one or the other was lost. Indeed, the diatom *Thalassiosira* bears both *EFL* and *eEF1A* genes [129] and might be an example of this situation. It is also possible that there was a single gain of EFL early in evolution followed by differential loss of it [124, 128, 129, 131, 132]. So far, EFL genes have been identified in widespread taxa, including diatoms, green and red algae, fungi, euglenozoans, foraminiferans, cryptophytes, goniomonads, katablepharid, chlorarachniophytes, oomycetes, dinoflagellates, choanozoans, centrohelids, and haptophytes [124–132]. Most of them are unicellular organisms. In contrast, eEF1A is found in most eukaryotes, and multiple copies of this gene have been found in some insect orders, including Coleoptera, Hymenoptera, Diptera, Thysanoptera, and Hemiptera [133].

 The eEF1A activity is modulated by diverse post-translational modifications, including phosphorylation, lysine methylation, and methyl-esterification. eEF1A also undergoes modification by covalent binding of ethanolamine phosphoglycerol (EPG), whose function is not known and for whom the number of moieties attached varies in different eukaryotes [134]. Moreover, in addition to its role in translation, eEF1A has been reported to play several “moonlighting” functions, including binding to cytoskeletal proteins, signal transduction, protein nuclear export and import of tRNAs into mitochondria [134]. It is not known whether EFL undergoes the same posttranslational modifications as eEF1A does and whether it also displays non-translational activities.

## 6. Divergence in the Termination Step

The termination of protein synthesis is governed by eRF1, which is a monophyletic and highly conserved protein that is universally present in eukaryotes. Comprehensive analyses of genomic datasets show that eRF1 was inherited by eukaryotes from archaeal ancestors and that most eukaryotes encode only one eRF1. Known exceptions are *Arabidopsis thaliana*, which possesses three *eRF1* genes, and the ciliates *Tetrahymena, Oxytricha, Nyctotherus, Oxytricha*, *Euplotes,* and *Paramecium* which have two *eRF1* genes [135–138]. Interestingly, unusually high rates of eRF1 evolution have been found in organisms with variant genetic codes, especially in the N-terminal domain, which is responsible for stop-codon recognition [30, 34, 135, 136, 138, 139]. eRF1 displays structural similarity to tRNA molecules and mimics its activity during binding of ribosomal A-site during recognition of a stop codon [34, 139–141]. Since mutations in eRF1 N-terminal domain switch from omnipotent to bipotent mode for stop-codon specificity [35–38, 141], most likely the accelerated evolution of eRF1 in organisms with variations to the nuclear genetic code has been driven mainly to accommodate these variations [30, 31, 34–38, 135, 138–141].

eRF3 is a GTPase that stimulates the activity of eRF1 during the translation termination process. eRF3 arose in early eukaryotes by the duplication of the GTPase eEF1A. Consistent with this, eRF3 binds and transports eRF1, a structural mimic of tRNA, to the ribosomal A-site, similar to the role of eEF1A in binding and delivering aminoacyl-tRNAs to the same site during translation elongation [142, 143]. eRF3 is much more divergent than eRF1, especially in its N-terminal domain. In addition, *eRF3 *is universal among eukaryotes, and most organisms only contain single-copies of this gene [137, 143]. In contrast, mammalian species express two eRF3s (viz. eRF3a and eRF3b; Figure 2). They possess different N regions and display drastically different tissue distribution and expression profiles during the cell cycle [143, 144]. Moreover, eRF3b but not eRF3a can substitute for yeast eRF3 in translation termination [145]. These observations indicate duplication and further functional divergence of eRF3 proteins in this lineage.

## 7. Divergence in the Recycling Step

Ribosomes from all eukaryotes perform elongation with eEF1A and eEF2. Interestingly, it has been known for some time that the yeast *Saccharomyces cerevisiae* requires an additional essential factor, eEF3, for the elongation cycle to proceed [146]. Genes encoding eEF3 were subsequently identified exclusively in other fungi (both yeasts and filamentous), including *Candida*, *Pneumocystis*, *Neurospora*, *Aspergillus,* and *Mucor* [147–150] (Figure 2). eEF3 is an ATPase that interacts with both ribosomal subunits and stimulates binding of aminoacyl-tRNA to the ribosomal A-site by enhancing the rate of deacylated tRNA dissociation from the E-site. Because E-site release is needed for efficient A-site binding of aminoacyl-tRNA, it was thought that eEF3 functions as a so-called “E-site” factor [16, 151]. Most recently, it was shown that post-termination complex, consisting of a ribosome, mRNA, and tRNA, is disassembled into single components by ATP and eEF3. Because the release of mRNA and deacylated tRNA and ribosome dissociation takes place simultaneously and no 40S—mRNA complexes remain, it is proposed that eEF3 activity promotes ribosome recycling [152]. “What were the evolutionary forces that led to the emergence of eEF3 exclusively in fungi?” is a very interesting, still open question.

## 8. Concluding Remarks

One of the most conspicuous features of life is its prominent ability to diversify. Current assessments of the biodiversity on Earth reaches 2 million species, although the true number of living organisms could easily be four times that number and likely much higher [153, 154]. The diversification of life has occurred at different levels, including the occupancy of ecological niches, behavioral patterns, body plans, and organismal complexity, and metabolic needs and capabilities. More recently, intensive whole-genome shotgun sequencing of microbial communities from different environments has unveiled a vast profusion of diversification also at the genetic level [155–157]. We have discussed that diversity also exists in the machinery that performs a fundamental process, translation, across eukaryotes. We speculate that the molecular diversification of the translation apparatus is among the basis that provided to early eukaryotes the scope to invade new ecological niches and overcome the different environmental and biological challenges this represented. Different evolutionary mechanisms might have been the driving forces leading to this molecular diversification in different lineages, including natural selection, sexual selection, genetic drift and neutral evolution. However, at this point, we can be nothing but speculative on the biological meaning of the molecular diversification reviewed here.

Traditional studies on so-called model organisms have taught us the global processes of eukaryotic translation. In the last years, the use of modern genome-wide, high-throughput technologies to study many non-model eukaryotes from different taxa has unveiled that diversification of the translation machinery configuration is far more expansive than previously thought. Collectively, these studies show that the translation apparatus in eukaryotes is far from being evolutionarily static. Therefore, we anticipate that, as more organisms are studied, additional diversification of components of the translation apparatus will be revealed. We believe that a better understanding of the diversity of all levels of organism will provide us a more profound understanding of Life.

## Figures and Tables

**Table 1:** Specialized activities of eIF4 proteins.

Protein^a^	Activity	Reference
eIF4E cognates		
Dm eIF4E-1, M eIF4E-1, Ce IFE-3, Sp eIF4E-1, Sc eIF4E, Plant eIF4E and eIF(iso)4E, Z eIF4E-1A, Gl eIF4E-2; Tb eIF4E-3 and eIF4E-4; Lm eIF4E-1 and eIF4E-4	Supports general cap-dependent initiation of translation. Essential gene.	[49, 54, 55, 57, 58, 62, 64, 65, 67, 72, 81–84]
M eIF4E-1	mRNA nucleocytoplasm transport.	[85]
Dm eIF4E-1	Involved in *sex-lethal* (*Sxl*)-dependent female-specific alternative splicing of male specific lethal-2 (msl-2) mRNA and *Sxl* pre-mRNAs.	[86]
Sp eIF4E-2	Supports cap-dependent translation initiation during stress response.	[62]
Ce IFE-1	Required for gametogenesis.	[87–89]
Ce IFE-2	Involved in chromosome segregation at meiosis at elevated temperatures.	[90]
Ce IFE-4	Promotes expression of specific mRNAs involved in egg lying. Nonessential gene.	[74]
Dm eIF4E-3	Testis-specific protein, essential for spermatogenesis.	[91]
La eF4E-4	Supports translation in promastigotes stage.	[71]
Dm 4E-HP, M 4E-HP	Negative regulator of translation.	[58, 73, 77, 78]
Xl eIF4E-1B	Negative regulator of translation.	[57, 79]
La eIF4E-1	Represses translation under heat shock conditions.	[71]
Gl eIF4E-1	Involved in nuclear snRNAs metabolism and play no role in translation.	[64]
Tb eIF4E-1 and eIF4E-2	Essential genes. Play no role in translation.	[49]
eIF4G cognates		
M eIF4G-I and eIF4G-II, Dm eIF4G, Sc eIF4G-I and eIF4G-II, plant eIF4G and Plant eIF(iso)4G, Ce p170 of IFG-1,	Scaffold protein. Supports general cap- and IRES-dependent initiation of translation.	[55, 60, 67, 71, 92–96]
Dm eIF4G-2	Support translation initiation in testis.	[47, 48]
M eIF4G-2	Involved in hematopoietic cell differentiation.	[97]
M eIF4G-3	Essential for spermatogenesis.	[98]
Ce IFG-1	p130 of *ifg-1* gene is involved in mitotic and early meiotic germ cell development.	[93]
La eIF4G-3	Supports translation in promastigotes stage.	[71]

^a^At, *Arabidopsis thaliana*; Ce, *Caenorhabditis elagans*; Dm, *Drosophila melanogaster*; Lm, *Leishmania major*; La, *Leishmania amazonensis*; M, *mammalian*; Nt, *N. tabacum*; Sc, *Saccharomyces cerevisiae*; Sp, *Schizosaccharomyces pombe*; W, *wheat germ*; Xl, *Xenopus laevis*; Z, *zebra fish*; Gl, *Giardia lamblia*; Tb, *Trypanosoma brusei*.

## References

[1] Mathews MB, Sonenberg N, Hershey JWB, Sonenberg N, Hershey JWB, Mathews MB (2000). Origins and principles of translational control. *Translational Control of Gene Expression*.

[2] Mathews MB, Sonenberg N, Hershey JWB, Mathews MB, Sonenberg N, Hershey JWB (2007). Origins and principles of translational control. *Translational Control in Biology and Medicine*.

[3] Hernández G, Altmann M, Lasko P (2010). Origins and evolution of the mechanisms regulating translation initiation in eukaryotes. *Trends in Biochemical Sciences*.

[4] Mazumder B, Seshadri V, Fox PL (2003). Translational control by the 3′-UTR: the ends specify the means. *Trends in Biochemical Sciences*.

[5] Renkawitz-Pohl R, Hempel L, Hollman M, Schafer MA, Gilbert LI, Iatrou K, Gill SS (2005). Spermatogenesis. *Comprehensive Molecular Insect Science*.

[6] Richter JD, Lasko P (2011). Translational control in oocyte development. *Cold Spring Harbor Perspectives in Biology*.

[7] Lasko P, Hershey JWB (2009). Translational control during early development. *Progress in Molecular Biology and Translational Science*.

[8] Sonenberg N, Hinnebusch AG (2007). New modes of translation control in development, behavior, and disease. *Molecular Cell*.

[9] Thompson B, Wickens M, Kimble J, Mathews MB, Sonenberg N, Hershey JWB (2007). Translational control in development. *Translational Control in Biology and Medicine*.

[10] Costa-Mattioli M, Sossin WS, Klann E, Sonenberg N (2009). Translational control of long-lasting synaptic plasticity and memory. *Neuron*.

[11] Schwanhüusser B, Busse D, Li N (2011). Global quantification of mammalian gene expression control. *Nature*.

[12] Vogel C, De Sousa Abreu R, Ko D (2010). Sequence signatures and mRNA concentration can explain two-thirds of protein abundance variation in a human cell line. *Molecular Systems Biology*.

[13] Mathews MB, Sonenberg N, Hershey JWB (2007). *Translational Bontrol in Biology and Medicine*.

[14] Sonenberg N, Hershey JWB, Mathews MB *Translational Control of Gene Expression*.

[15] Hershey JWB, Merrick WC, Sonenberg N, Hershey JWB, Mathews MB (2000). Pathway and mechanism of initiation of protein synthesis. *Translational Control of Gene Expression*.

[16] Kapp LD, Lorsch JR (2004). The molecular mechanics of eukaryotic translation. *Annual Review of Biochemistry*.

[17] Sonenberg N, Hinnebusch AG (2009). Regulation of translation initiation in eukaryotes: mechanisms and biological targets. *Cell*.

[18] Jackson RJ, Hellen CUT, Pestova TV (2010). The mechanism of eukaryotic translation initiation and principles of its regulation. *Nature Reviews Molecular Cell Biology*.

[19] Preiss T, Hentze MW (2003). Starting the protein synthesis machine: eukaryotic translation initiation. *BioEssays*.

[20] Gebauer F, Hentze MW (2004). Molecular mechanisms of translational control. *Nature Reviews Molecular Cell Biology*.

[21] Dounda JA, Sarnow P, Mathews MB, Sonenberg N, Hershey JWB (2007). Translation initiation by viral internal ribosome entry sites. *Translational Control in Biology and Medicine*.

[22] Hernández G (2008). Was the initiation of translation in early eukaryotes IRES-driven?. *Trends in Biochemical Sciences*.

[23] Pacheco A, Martinez-Salas E (2010). Insights into the biology of IRES elements through riboproteomic approaches.. *Journal of Biomedicine and Biotechnology*.

[24] Taylor DJ, Frank J, Kinzy TG, Mathews MB, Sonenberg N, Hershey JWB (2007). Structure and function of the eukaryotic ribosome and elongation factors. *Translational Control in Biology and Medicine*.

[25] Andersen GR, Nissen P, Nyborg J (2003). Elongation factors in protein biosynthesis. *Trends in Biochemical Sciences*.

[26] Herbert TP, Proud CG, Mathews MB, Sonenberg N, Hershey JWB (2007). Regulation of translation elongation and the cotranslational protein targeting pathway. *Translational Control in Biology and Medicine*.

[27] Ehrenberg M, Hauryliuk V, Crist CG, Nakamura Y, Mathews MB, Sonenberg N, Hershey JWB (2007). Translation termination, the prion [PSI+], and ribosomal recycling. *Translational Control in Biology and Medicine*.

[28] Jackson RJ, Hellen CUT, Pestova TV (2012). Termination and post-termination events in eukaryotic translation. *Advances in Protein Chemistry and Structural Biology*.

[29] Szymański M, Barciszewski J (2007). The genetic code—40 years on. *Acta Biochimica Polonica*.

[30] Knight RD, Freeland SJ, Landweber LF (2001). Rewiring the keyboard: evolvability of the genetic code. *Nature Reviews Genetics*.

[31] Lobanov AV, Turanov AA, Hatfield DL, Gladyshev VN (2010). Dual functions of codons in the genetic code. *Critical Reviews in Biochemistry and Molecular Biology*.

[32] Koonin EV, Novozhilov AS (2009). Origin and evolution of the genetic code: the universal enigma. *IUBMB Life*.

[33] Santos MAS, Moura G, Massey SE, Tuite MF (2004). Driving change: the evolution of alternative genetic codes. *Trends in Genetics*.

[34] Lozupone CA, Knight RD, Landweber LF (2001). The molecular basis of nuclear genetic code change in ciliates. *Current Biology*.

[35] Eliseev B, Kryuchkova P, Alkalaeva E, Frolova L (2011). A single amino acid change of translation termination factor eRF1 switches between bipotent and omnipotent stop-codon specificity. *Nucleic Acids Research*.

[36] Lekomtsev S, Kolosov P, Bidou L, Frolova L, Rousset JP, Kisselev L (2007). Different modes of stop codon restriction by the Stylonychia and Paramecium eRF1 translation termination factors. *Proceedings of the National Academy of Sciences of the United States of America*.

[37] Inagaki Y, Blouin C, Doolittle WF, Roger AJ (2002). Convergence and constraint in eukaryotic release factor 1 (eRF1) domain 1: the evolution of stop codon specificity. *Nucleic Acids Research*.

[38] Seit-Nebi A, Frolova L, Kisselev L (2002). Conversion of omnipotent translation termination factor eRF1 into ciliate-like UGA-only unipotent eRF1. *EMBO Reports*.

[39] Cocquyt E, Gile GH, Leliaert F, Verbruggen H, Keeling PJ, De Clerck O (2010). Complex phylogenetic distribution of a non-canonical genetic code in green algae. *BMC Evolutionary Biology*.

[40] Hernández G (2009). On the origin of the cap-dependent initiation of translation in eukaryotes. *Trends in Biochemical Sciences*.

[41] Kyrpides NC, Woese CR (1998). Universally conserved translation initiation factors. *Proceedings of the National Academy of Sciences of the United States of America*.

[42] Londei P (2005). Evolution of translational initiation: new insights from the archaea. *FEMS Microbiology Reviews*.

[43] Aravind L, Koonin EV (2000). Eukaryote-specific domains in translation initiation factors: implications for translation regulation and evolution of the translation system. *Genome Research*.

[44] Benelli D, Londei P (2009). Begin at the beginning: evolution of translational initiation. *Research in Microbiology*.

[45] Hernández G, Vazquez-Pianzola P (2005). Functional diversity of the eukaryotic translation initiation factors belonging to eIF4 families. *Mechanisms of Development*.

[46] Joshi B, Lee K, Maeder DL, Jagus R (2005). Phylogenetic analysis of eIF4E-family members. *BMC Evolutionary Biology*.

[47] Baker CC, Fuller MT (2007). Translational control of meiotic cell cycle progression and spermatid differentiation in male germ cells by a novel elF4G homolog. *Development*.

[48] Franklin-Dumont TM, Chatterjee C, Wasserman SA, DiNardo S (2007). A novel elF4G homolog, off-schedule, couples translational control to meiosis and differentiation in Drosophila spermatocytes. *Development*.

[49] Freire ER, Dhalia R, Moura DMN (2011). The four trypanosomatid eIF4E homologues fall into two separate groups, with distinct features in primary sequence and biological properties. *Molecular and Biochemical Parasitology*.

[50] Keiper BD, Lamphear BJ, Deshpande AM (2000). Functional characterization of five eIF4E isoforms in Caenorhabditis elegans. *Journal of Biological Chemistry*.

[51] Miyoshi H, Dwyer DS, Keiper BD, Jankowska-Anyszka M, Darzynkiewicz E, Rhoads RE (2002). Discrimination between mono- and trimethylated cap structures by two isoforms of Caenorhabditis elegans eIF4E. *EMBO Journal*.

[52] Lasko P (2000). The Drosophila melanogaster genome: translation factors and RNA binding proteins. *Journal of Cell Biology*.

[53] Morales J, Mulner-Lorillon O, Cosson B (2006). Translational control genes in the sea urchin genome. *Developmental Biology*.

[54] Jankowska-Anyszka M, Lamphear BJ, Aamodt EJ (1998). Multiple isoforms of eukaryotic protein synthesis initiation factor 4E in Caenorhabditis elegans can distinguish between mono- and trimethylated mRNA cap structures. *Journal of Biological Chemistry*.

[55] Browning KS (2004). Plant translation initiation factors: it is not easy to be green. *Biochemical Society Transactions*.

[56] Mayberry LK, Leah Allen M, Dennis MD, Browning KS (2009). Evidence for variation in the optimal translation initiation complex: plant eIF4B, eIF4F, and eIF(iso)4F differentially promote translation of mRNAs. *Plant Physiology*.

[57] Robalino J, Joshi B, Fahrenkrug SC, Jagus R (2004). Two zebrafish eIF4E family members are differentially expressed and functionally divergent. *Journal of Biological Chemistry*.

[58] Hernández G, Altmann M, Sierra JM (2005). Functional analysis of seven genes encoding eight translation initiation factor 4E (eIF4E) isoforms in Drosophila. *Mechanisms of Development*.

[59] Evsikov AV, Marín de Evsikova C (2009). Evolutionary origin and phylogenetic analysis of the novel oocyte-specific eukaryotic translation initiation factor 4E in Tetrapoda. *Development Genes and Evolution*.

[60] Goyer C, Altmann M, Lee HS (1993). TIF4631 and TIF4632: two yeast genes encoding the high-molecular-weight subunits of the cap-binding protein complex (eukaryotic initiation factor 4F) contain an RNA recognition motif-like sequence and carry out an essential function. *Molecular and Cellular Biology*.

[61] Wakiyama M, Suzuki A, Saigoh M (2001). Analysis of the isoform of Xenopus euakryotic translation initiation factor 4E. *Bioscience, Biotechnology and Biochemistry*.

[62] Ptushkina M, Berthelot K, Von der Haar T, Geffers L, Warwicker J, McCarthy JEG (2001). A second elF4E protein in Schizosaccharomyces pombe has distinct elF4G-binding properties. *Nucleic Acids Research*.

[63] Joshi B, Cameron A, Jagus R (2004). Characterization of mammalian eIF4E-family members. *European Journal of Biochemistry*.

[64] Li L, Wang CC (2005). Identification in the ancient protist Giardia lamblia of two eukaryotic translation initiation factor 4E homologues with distinctive functions. *Eukaryotic Cell*.

[65] Yoffe Y, Zuberek J, Lerer A (2006). Binding specificities and potential roles of isoforms of eukaryotic initiation factor 4E in Leishmania. *Eukaryotic Cell*.

[66] Dhalia R, Reis CRS, Freire ER (2005). Translation initiation in Leishmania major: characterisation of multiple eIF4F subunit homologues. *Molecular and Biochemical Parasitology*.

[67] Gallie DR, Mathews MB, Sonenberg N, Hershey JWB (2007). Translational control in plants and chloroplasts. *Translational Control in Biology and Medicine*.

[68] Hernández G, Vázques-Pianzola P, Zurbriggen A, Altmann M, Sierra JM, Rivera-Pomar R (2004). Two functionally redundant isoforms of Drosophila melanogaster eukaryotic initiation factor 4B are involved in cap-dependent translation, cell survival, and proliferation. *European Journal of Biochemistry*.

[69] Hernández G, Del Mar Castellano M, Agudo M, Sierra JM (1998). Isolation and characterization of the cDNA and the gene for eukaryotic translation initiation factor 4G from Drosophila melanogaster. *European Journal of Biochemistry*.

[70] Owttrim GW, Mandel T, Trachsel H, Thomas AA, Kuhlemeier C (1994). Characterization of the tobacco eIF-4A gene family. *Plant Molecular Biology*.

[71] Zinoviev A, Leger M, Wagner G, Shapira M (2011). A novel 4E-interacting protein in Leishmania is involved in stage-specific translation pathways. *Nucleic Acids Research*.

[72] Yoffe Y, Léger M, Zinoviev A (2009). Evolutionary changes in the Leishmania eIF4F complex involve variations in the eIF4E-eIF4G interactions. *Nucleic Acids Research*.

[73] Cho PF, Poulin F, Cho-Park YA (2005). A new paradigm for translational control: inhibition via 5′-3′ mRNA tethering by Bicoid and the eIF4E cognate 4EHP. *Cell*.

[74] Dinkova TD, Keiper BD, Korneeva NL, Aamodt EJ, Rhoads RE (2005). Translation of a small subset of Caenorhabditis elegans mRNAs is dependent on a specific eukaryotic translation initiation factor 4E isoform. *Molecular and Cellular Biology*.

[75] Rhoads RE (2009). EIF4E: new family members, new binding partners, new roles. *Journal of Biological Chemistry*.

[76] Ruud KA, Kuhlow C, Goss DJ, Browning KS (1998). Identification and characterization of a novel cap-binding protein from Arabidopsis thaliana. *Journal of Biological Chemistry*.

[77] Rom E, Kim HC, Gingras AC (1998). Cloning and characterization of 4EHP, a novel mammalian eIF4E-related cap-binding protein. *Journal of Biological Chemistry*.

[78] Villaescusa JC, Buratti C, Penkov D (2009). Cytoplasmic Prep1 interacts with 4EHP inhibiting Hoxb4 translation. *PLoS One*.

[79] Minshall N, Reiter MH, Weil D, Standart N (2007). CPEB interacts with an ovary-specific eIF4E and 4E-T in early Xenopus oocytes. *Journal of Biological Chemistry*.

[80] Lall S, Friedman CC, Jankowska-Anyszka M, Stepinski J, Darzynkiewicz E, Davis RE (2004). Contribution of trans-splicing, 5′-leader length, cap-poly(A) synergism, and initiation factors to nematode translation in an Ascaris suum embryo cell-free system. *Journal of Biological Chemistry*.

[81] Altmann M, Handschin C, Trachsel H (1987). mRNA cap-binding protein: cloning of the gene encoding protein synthesis initiation factor eIF-4E from Saccharomyces cerevisiae. *Molecular and Cellular Biology*.

[82] Maroto FG, Sierra JM (1989). Purification and characterization of mRNA cap-binding protein from Drosophila melanogaster embryos. *Molecular and Cellular Biology*.

[83] Rodriguez CM, Freire MA, Camilleri C, Robaglia C (1998). The Arabidosis thaliana cDNAs encoding for eIF4E and eIF(iso)4E are not functionally equivalent for yeast complementation and are differentially expressed during plant development. *Plant Journal*.

[84] Sonenberg N (2008). eIF4E, the mRNA cap-binding protein: from basic discovery to translational research. *Biochemistry and Cell Biology*.

[85] Rong L, Livingstone M, Sukarieh R (2008). Control of eIF4E cellular localization by eIF4E-binding proteins, 4E-BPs. *RNA*.

[86] Graham PL, Yanowitz JL, Penn JKM, Deshpande G, Schedl P (2011). The translation initiation factor eif4e regulates the Sex-Specific expression of the master switch gene Sxl in Drosophila melanogaster. *PLoS Genetics*.

[87] Amiri A, Keiper BD, Kawasaki I (2001). An isoform of elF4E is a component of germ granules and is required for spermatogenesis in *C. elegans*. *Development*.

[88] Henderson MA, Croniand E, Dunkelbarger S, Contreras V, Strome S, Keiper BD (2009). A germline-specific isoform of eIF4E (IFE-1) is required for efficient translation of stored mRNAs and maturation of both oocytes and sperm. *Journal of Cell Science*.

[89] Kawasaki I, Jeong MH, Shim YH (2011). Regulation of sperm-specific proteins by IFE-1, a germline-specific homolog of eIF4E, in C. elegans. *Molecules and Cells*.

[90] Song A, Labella S, Korneeva NL (2010). A *C. elegans* eIF4E-family member upregulates translation at elevated temperatures of mRNAs encoding MSH-5 and other meiotic crossover proteins. *Journal of Cell Science*.

[91] Hernández G, Gandin V, Han H, Ferreira T, Sonenberg N, Lasko P Translational control by Drosophila eIF4E-3 is essential for cell differentiation during spermiogenesis.

[92] Zapata JM, Martinez MA, Sierra JM (1994). Purification and characterization of eukaryotic polypeptide chain initiation factor 4F from Drosophila melanogaster embryos. *Journal of Biological Chemistry*.

[93] Contreras V, Richardson MA, Hao E, Keiper BD (2008). Depletion of the cap-associated isoform of translation factor eIF4G induces germline apoptosis in *C. elegans*. *Cell Death and Differentiation*.

[94] Prévôt D, Darlix JL, Ohlmann T (2003). Conducting the initiation of protein synthesis: the role of eIF4G. *Biology of the Cell*.

[95] Pestova TV, Shatsky IN, Hellen CUT (1996). Functional dissection of eukaryotic initiation factor 4F: the 4A subunit and the central domain of the 4G subunit are sufficient to mediate internal entry of 43S preinitiation complexes. *Molecular and Cellular Biology*.

[96] Gradi A, Imataka H, Svitkin YV (1998). A novel functional human eukaryotic translation initiation factor 4G. *Molecular and Cellular Biology*.

[97] Caron S, Charon M, Cramer E, Sonenberg N, Dusanter-Fourt I (2004). Selective modification of eukaryotic initiation factor 4F (eIF4F) at the onset of cell differentiation: recruitment of eIF4GII and long-lasting phosphorylation of eIF4E. *Molecular and Cellular Biology*.

[98] Sun F, Palmer K, Handel MA (2010). Mutation of Eif4g3, encoding a eukaryotic translation initiation factor, causes male infertility and meiotic arrest of mouse spermatocytes. *Development*.

[99] Hinnebusch AG (2006). eIF3: a versatile scaffold for translation initiation complexes. *Trends in Biochemical Sciences*.

[100] Guo J, Jin Z, Yang X, Li JF, Chen JG (2011). Eukaryotic initiation factor 6, an evolutionarily conserved regulator of ribosome biogenesis and protein translation. *Plant Signaling and Behavior*.

[101] Stevenson AL, McCarthy JEG (2008). Found in translation: another RNA helicase function. *Molecular Cell*.

[102] Parsyan A, Svitkin Y, Shahbazian D (2011). MRNA helicases: the tacticians of translational control. *Nature Reviews Molecular Cell Biology*.

[103] Linder P, Jankowsky E (2011). From unwinding to clamping—the DEAD box RNA helicase family. *Nature Reviews Molecular Cell Biology*.

[104] Weston A, Sommerville J (2006). Xp54 and related (DDX6-like) RNA helicases: roles in messenger RNP assembly, translation regulation and RNA degradation. *Nucleic Acids Research*.

[105] Bush MS, Hutchins AP, Jones AME (2009). Selective recruitment of proteins to 5′ cap complexes during the growth cycle in Arabidopsis. *Plant Journal*.

[106] Richter JD, Sonenberg N (2005). Regulation of cap-dependent translation by eIF4E inhibitory proteins. *Nature*.

[107] Proud CG (2007). Signalling to translation: how signal transduction pathways control the protein synthetic machinery. *Biochemical Journal*.

[108] Raught B, Gingras AC, Mathews MB, Sonenberg N, Hershey JWB (2007). Signaling to translation initiation. *Translational Control in Biology and Medicine*.

[109] Furic L, Rong L, Larsson O (2010). EIF4E phosphorylation promotes tumorigenesis and is associated with prostate cancer progression. *Proceedings of the National Academy of Sciences of the United States of America*.

[110] Scheper GC, Proud CG (2002). Does phosphorylation of the cap-binding protein eIF4E play a role in translation initiation?. *European Journal of Biochemistry*.

[111] Buxade M, Parra-Palau JL, Proud CG (2008). The Mnks: MAP kinase-interacting kinases (MAP kinase signal-integrating kinases).. *Frontiers in Bioscience*.

[112] Reiling JH, Doepfner KT, Hafen E, Stocker H (2005). Diet-dependent effects of the Drosophila Mnk1/Mnk2 homolog Lk6 on growth via eIF4E. *Current Biology*.

[113] Lachance PED, Miron M, Raught B, Sonenberg N, Lasko P (2002). Phosphorylation of eukaryotic translation initiation factor 4E is critical for growth. *Molecular and Cellular Biology*.

[114] Arquier N, Bourouis M, Colombani J, Léopold P (2005). Drosophila Lk6 kinase controls phosphorylation of eukaryotic translation initiation factor 4E and promotes normal growth and development. *Current Biology*.

[115] Parra-Palau JL, Scheper GC, Harper DE, Proud CG (2005). The Drosophila protein kinase LK6 is regulated by ERK and phosphorylates the eukaryotic initiation factor eIF4E in vivo. *Biochemical Journal*.

[116] Nett IRE, Martin DMA, Miranda-Saavedra D (2009). The phosphoproteome of bloodstream form Trypanosoma brucei, causative agent of African sleeping sickness. *Molecular and Cellular Proteomics*.

[117] Zanchin NIT, McCarthy JEG (1995). Characterization of the in vivo phosphorylation sites of the mRNA*·*Cap- binding complex proteins eukaryotic initiation factor-4E and p20 in Saccharomyces cerevisiae. *Journal of Biological Chemistry*.

[118] Dever TE, Dar AC, Sicheri F, Mathews MB, Sonenberg N, Hershey JWB (2007). The eIF2a kinases. *Translational Control in Biology and Medicine*.

[119] Rothenburg S, Deigendesch N, Dey M, Dever TE, Tazi L (2008). Double-stranded RNA-activated protein kinase PKR of fishes and amphibians: varying the number of double-stranded RNA binding domains and lineage-specific duplications.. *BMC biology*.

[120] Szymański M, Deniziak M, Barciszewski J (2000). The new aspects of aminoacyl-tRNA synthetases. *Acta Biochimica Polonica*.

[121] Guo M, Yang XL, Schimmel P (2010). New functions of aminoacyl-tRNA synthetases beyond translation. *Nature Reviews Molecular Cell Biology*.

[122] Ray PS, Sullivan JC, Jia J, Francis J, Finnerty JR, Fox PL (2011). Evolution of function of a fused metazoan tRNA synthetase. *Molecular Biology and Evolution*.

[123] Fox PL, Ray PS, Arif A, Jia J, Mathews MB, Sonenberg N, Hershey JWB (2007). Noncanonical functions of aminoacyl-tRNA synthetases in translational control. *Translational Control in Biology and Medicine*.

[124] Keeling PJ, Inagaki Y (2004). A class of eukaryotic GTPase with a punctate distribution suggesting multiple functional replacements of translation elongation factor 1*α*. *Proceedings of the National Academy of Sciences of the United States of America*.

[125] Sakaguchi M, Takishita K, Matsumoto T, Hashimoto T, Inagaki Y (2009). Tracing back EFL gene evolution in the cryptomonads-haptophytes assemblage: separate origins of EFL genes in haptophytes, photosynthetic cryptomonads, and goniomonads. *Gene*.

[126] Gile GH, Novis PM, Cragg DS, Zuccarello GC, Keeling PJ (2009). The distribution of elongation factor-1 alpha (EF-1*α*), elongation factor-like (EFL), and a non-canonical genetic code in the ulvophyceae: Discrete genetic characters support a consistent phylogenetic framework. *Journal of Eukaryotic Microbiology*.

[127] Cocquyt E, Verbruggen H, Leliaert F, Zechman FW, Sabbe K, De Clerck O (2009). Gain and loss of elongation factor genes in green algae. *BMC Evolutionary Biology*.

[128] Noble GP, Rogers MB, Keeling PJ (2007). Complex distribution of EFL and EF-1*α* proteins in the green algal lineage. *BMC Evolutionary Biology*.

[129] Kamikawa R, Inagaki Y, Sako Y (2008). Direct phylogenetic evidence for lateral transfer of elongation factor-like gene. *Proceedings of the National Academy of Sciences of the United States of America*.

[130] Kamikawa R, Yabuki A, Nakayama T, Ishida KI, Hashimoto T, Inagaki Y (2011). Cercozoa comprises both EF-1*α*-containing and EFL-containing members. *European Journal of Protistology*.

[131] Kamikawa R, Sakaguchi M, Matsumoto T, Hashimoto T, Inagaki Y (2010). Rooting for the root of elongation factor-like protein phylogeny. *Molecular Phylogenetics and Evolution*.

[132] Gile GH, Faktorová D, Castlejohn CA (2009). Distribution and phylogeny of EFL and EF1alpha in Euglenozoa suggest ancestral co-occurrence followed by differential loss. *PLoS One*.

[133] Djernaes M, Damgaard J (2006). Exon-intron structure, paralogy and sequenced regions of elongation factor-1 alpha in Hexapoda. *Arthropod Systematics and Phylogeny*.

[134] Greganova E, Altmann M, Bütikofer P (2011). Unique modifications of translation elongation factors. *FEBS Journal*.

[135] Kim OTP, Yura K, Go N, Harumoto T (2005). Newly sequenced eRF1s from ciliates: the diversity of stop codon usage and the molecular surfaces that are important for stop codon interactions. *Gene*.

[136] Moreira D, Kervestin S, Jean-Jean O, Philippe H (2002). Evolution of eukaryotic translation elongation and termination factors: variations of evolutionary rate and genetic code deviations. *Molecular Biology and Evolution*.

[137] Atkinson GC, Baldauf SL, Hauryliuk V (2008). Evolution of nonstop, no-go and nonsense-mediated mRNA decay and their termination factor-derived components. *BMC Evolutionary Biology*.

[138] Inagaki Y, Doolittle WF (2001). Class I release factors in ciliates with variant genetic codes. *Nucleic Acids Research*.

[139] Song H, Mugnier P, Das AK (2000). The crystal structure of human eukaryotic release factor eRF1—mechanism of stop codon recognition and peptidyl-tRNA hydrolysis. *Cell*.

[140] Kolosov P, Frolova L, Seit-Nebi A (2005). Invariant amino acids essential for decoding function of polypeptide release factor eRF1. *Nucleic Acids Research*.

[141] Ito K, Frolova L, Seit-Nebi A, Karamyshev A, Kisselev L, Nakamura Y (2002). Omnipotent decoding potential resides in eukaryotic translation termination factor eRF1 of variant-code organisms and is modulated by the interactions of amino acid sequences within domain 1. *Proceedings of the National Academy of Sciences of the United States of America*.

[142] Inagaki Y, Doolittle WF (2000). Evolution of the eukaryotic translation termination system: origins of release factors. *Molecular Biology and Evolution*.

[143] Zhouravleva G, Schepachev V, Petrova A, Tarasov O, Inge-Vechtomov S (2006). Evolution of translation termination factor eRF3: is GSPT2 generated by retrotransposition of GSPT1’s mRNA?. *IUBMB Life*.

[144] Hoshino SI, Imai M, Mizutani M (1998). Molecular cloning of a novel member of the eukaryotic polypeptide chain- releasing factors (eRF): its identification as eRF3 interacting with eRF1. *Journal of Biological Chemistry*.

[145] Goff CL, Zemlyanko O, Moskalenko S (2002). Mouse GSPT2, but not GSPT1, can substitute for yeast eRF3 in vivo. *Genes to Cells*.

[146] Skogerson L, Wakatama E (1976). A ribosome dependent GTPase from yeast distinct from elongation factor 2. *Proceedings of the National Academy of Sciences of the United States of America*.

[147] Ypma-Wong MF, Fonzi WA, Sypherd PS (1992). Fungus-specific translation elongation factor 3 gene present in *Pneumocystis carinii*. *Infection and Immunity*.

[148] Qin S, Xie A, Bonato MCM, McLaughlin CS (1990). Sequence analysis of the translational elongation factor 3 from Saccharomyces cerevisiae. *Journal of Biological Chemistry*.

[149] Di Domenico BJ, Lupisella J, Sandbaken M, Chakraburtty K (1992). Isolation and sequence analysis of the gene encoding translation elongation factor 3 from Candida albicans. *Yeast*.

[150] Skogerson L (1979). Separation and characterization of yeast elongation factors. *Methods in Enzymology*.

[151] Chakraburtty K, Triana-Alonso FJ (1998). Yeast elongation factor 3: structure and function. *Biological Chemistry*.

[152] Kurata S, Nielsen KH, Mitchell SF, Lorsch JR, Kaji A, Kaji H (2010). Ribosome recycling step in yeast cytoplasmic protein synthesis is catalyzed by eEF3 and ATP. *Proceedings of the National Academy of Sciences of the United States of America*.

[153] Bisby FA, Roskov YR, Orrell TM, Nicolson D, Paglinawan LE Species 2000 & ITIS Catalogue of Life: 2010 Annual Checklist. http://www.catalogueoflife.org/annual-checklist/2010.

[154] Mora C, Titterson DP, Adl S, Simpson AGB, Worm B (2011). How many species are there on Earth and in the ocean. *PLoS Biology*.

[155] Tringe SG, Von Mering C, Kobayashi A (2005). Comparative metagenomics of microbial communities. *Science*.

[156] McHardy AC, Rigoutsos I (2007). What’s in the mix: phylogenetic classification of metagenome sequence samples. *Current Opinion in Microbiology*.

[157] Venter JC, Remington K, Heidelberg JF (2004). Environmental genome shotgun sequencing of the Sargasso Sea. *Science*.

